# A Pacific carbonate budgets approach reveals overall net positive production on O‘ahu, Hawai‘i

**DOI:** 10.1371/journal.pone.0354186

**Published:** 2026-08-12

**Authors:** Candace E. Alagata, Hannah C. Barkley, Joy N. Smith, Ariel A. Halperin, Rebecca M. Weible, Walter C. Oechel

**Affiliations:** 1 Cooperative Institute for Marine and Atmospheric Research, University of Hawaiʻi, Honolulu, Hawaiʻi, United States of America; 2 Pacific Islands Fisheries Science Center, National Marine Fisheries Service, Honolulu, Hawaiʻi, United States of America; 3 Department of Biology, San Diego State University, San Diego, California, United States of America; MARE – Marine and Environmental Sciences Centre, PORTUGAL

## Abstract

As changes in regional and global climates continue to exacerbate natural and anthropogenic stressors on coral reef ecosystems, quantifying a reef’s ability to produce its calcium carbonate framework and identifying the taxa that drive calcification and bioerosion dynamics are essential for effective coral reef management. The net carbonate production rate, or carbonate budget, of a coral reef estimates the balance between calcium carbonate (CaCO_3_) production and erosion. Census-based methods estimate this budget as a single representative functional health metric, alongside additional reef health indicators such as coral cover, diversity, and structural complexity. Here, we report an assessment of census-based coral reef carbonate budgets for seven coral reef sites around Oʻahu, Hawaiʻi. Coral CaCO_3_ production rates were calculated using a regionally-refined database of species- and genera-specific calcification rates for corals within the Pacific Islands region. Additionally, we tested metrics of percent coral cover, coral genera diversity, and structural complexity (rugosity) as predictors for reef framework growth and erosion. Net CaCO_3_ production for Oʻahu ranged from −0.15 ± 0.34 to 6.34 ± 0.82 kg CaCO_3_ m^-2^ yr^-1^, with four sites demonstrating net positive production and three sites near zero net production. Coral cover and coral genera diversity were strong predictors of gross carbonate production, while reef rugosity was a poor predictor. The ratio of percent coral cover to carbonate production differed among coral taxa, suggesting that the use of species- and genus-specific growth rates may improve the representativeness of gross and net carbonate production calculations.

## Introduction

The ability of a coral reef to provide essential ecosystem services, food, and critical habitat for thousands of marine species relies on its physical framework [1–4]. Complex three-dimensional reef framework is built by the accumulation of calcium carbonate (CaCO_3_) over long periods of time and persists when coral and calcifier CaCO_3_ production exceeds the physical, chemical, and biological erosion of the reef framework [5–7]. However, the structural integrity of coral reef habitats is increasingly threatened by a multitude of direct and indirect anthropogenic disturbances [8–10]. Global climate change and ocean acidification, as well as localized stressors like land-based sources of pollution have already led to worldwide declines in coral cover, diversity, and complex reef structure and will continue to threaten reef persistence over the next several decades and likely much longer [11–15].

Understanding the accumulation and persistence of reef carbonate framework is critical for assessing current status and future trajectories of coral reef ecosystem health. Net carbonate production rates—a reef’s carbonate budget, calculated as the rate of gross CaCO_3_ production minus the rate of CaCO_3_ erosion [16,17]—are typically estimated using ecological census-based approaches, whereas geological techniques are more commonly used to quantify long-term reef accretion and carbonate deposition [18,19]. Census-based carbonate budget surveys, predominantly conducted using *ReefBudget* methodology [20], have become increasingly widespread over the past two decades [21]. These methodologies use ecological survey data on cover and abundance of individual carbonate producers (including scleractinian corals and calcareous encrusters) and eroders (including parrotfish, urchins, and macro- and micro-bioeroders), together with literature-derived CaCO_3_ gross production and erosion rates, to estimate the net carbonate production rate of a reef [17]. Carbonate budget estimates have been used globally as indicators for ecosystem degradation and to determine impacts of frequent thermal stress events on budget states [22–29]. However, census-based methods can be complex and time-intensive, limiting their application for many time-limited coral reef monitoring programs [21]. In lieu of carbonate budget data, metrics typically collected by monitoring efforts (such as coral percent cover, coral genera diversity, reef structural complexity, and bioeroder abundance) have often been used to assess ecological states of coral reefs under continued climatic stress. Generally, structurally complex reefs with high cover or abundance of habitat-altering taxa are associated with higher rates of production and erosion [25,30], with site-level differences linked to variability in both the composition and calcification/erosion rates of the taxa present [25,28,30,31]. Therefore, how these common metrics may relate, and potentially serve as predictors, for the growth and erosion of a reef’s physical framework is still poorly understood [32].

In the Pacific, several large-scale studies have assessed carbonate budgets for multiple reefs and island-systems [14,33,34]. Coral reefs around heavily populated islands like Oʻahu, Hawaiʻi have been frequently assessed using standard reef metrics of coral cover and diversity, yet few studies have quantified reef production and erosion. While reefs in the Hawaiian Archipelago have historically demonstrated relatively high resilience to global and local anthropogenic stressors [35–38], Hawaiʻi reefs may be approaching neutral or negative carbonate production rates under continued climatic and anthropogenic stress [34,39–42]. Accurately characterizing carbonate budget states requires regionally-specific coral growth and carbonate bioerosion rates. Current *ReefBudget* methodology [20] uses a genera and morphology-specific coral growth rates database for the broader Indo-Pacific region; yet, spatial variation in growth rates of individual coral taxa can occur across sub-regions due to variations in environmental conditions [43–47]. Restricting the database to coral growth rates found within the specific region in which they are being used to estimate coral reef carbonate production can provide more precise gross carbonate production calculations, and subsequently overall more precise net carbonate budget estimates [48]. However, regionally refining a coral growth rates database for assessment of reefs within the Pacific Islands region has yet to be accomplished.

Here we evaluate carbonate budget states for Hawaiʻi coral reefs using a regionally specific approach. Applying *ReefBudget* census-based methodologies and a refined species and genera-specific coral growth rates database for the Pacific Islands region, we aimed to: (1) determine whether coral reefs around Oʻahu are net producing or net eroding; (2) assess whether estimates of gross carbonate production differs when calculated using Pacific Islands coral growth rates compared to the *ReefBudget* coral growth rates derived from the broader Indo-Pacific; (3) identify which components of the net carbonate budget contribute most to gross carbonate production and bioerosion, and (4) evaluate whether standard ecological metrics (e.g., coral cover, coral genera diversity, structural complexity, bioeroder density, abundance, size and biomass) are reliable indicators of gross carbonate production and bioerosion on Oʻahu.

## Materials and methods

### Carbonate budget field surveys

**Fig 1:**
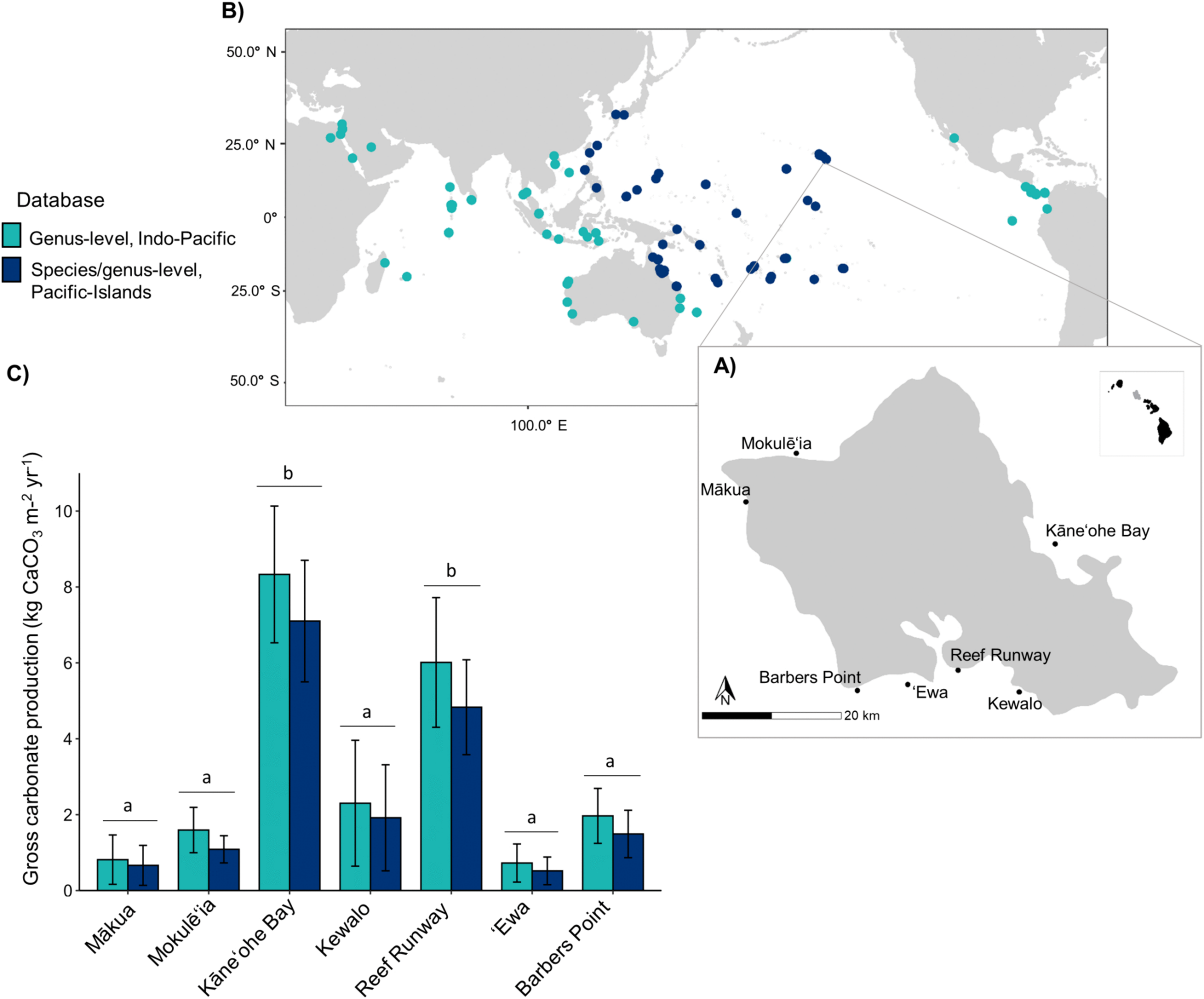
(A) Map of the seven coral reef survey sites around Oʻahu, Hawaiʻi. (B) Map of coral linear extension and skeletal density studies used in the ‘Genus-level, Indo-Pacific’ database (derived from *ReefBudget* [20]) or the refined ‘Species/genus-level, Pacific Islands’ database. (C) Mean gross carbonate production rates ± 95% confidence intervals (CI) estimated for the seven Oʻahu survey sites using both databases. Letters a and b indicate Tukey post hoc test results, where mean gross carbonate production estimates for sites with the letter a are similar to each other and significantly different from sites with the letter b. For all sites, mean gross carbonate production estimates were statistically similar using either database indicated by the line underscoring both letters. Note the ‘Genus-level, Indo-Pacific’ database also includes all studies from the ‘Species/genus-level, Pacific Islands’ database.

Carbonate budget field surveys were conducted in June and July, 2021 at seven sites around the island of Oʻahu, Hawaiʻi (Fig 1A). Survey sites were selected from NOAA’s National Coral Reef Monitoring Program’s (NCRMP) mid-depth (~9−15 m) fixed sites in fringing reef habitat. Sites were selected to represent a range of environmental conditions across the island. Sites Mokulēʻia and Mākua are located on the northwest coast, which are exposed to large north-northwestern Pacific swells (up to ≥ 7 m) in the winter [49]. The Kāneʻohe Bay survey site is located on the eastern (windward) coast on the forereef outside a large, semi-enclosed embayment, which modulates direct exposure to offshore swell [50]. Sites Kewalo, Reef Runway, ʻEwa, and Barbers Point are located on the relatively highly populated southern coast, which is exposed to southern Pacific summer swells (up to 3 m) [51]. Benthic, urchin, and fish surveys were conducted following the *ReefBudget* Indo-Pacific methodologies as described in Perry et al. [20] and Barkley et al. [52]. Field work was conducted under Special Activities Permit No. 2022−25 issued by the Department of Land and Natural Resources, Division of Aquatic Resources. As this field study was entirely observational and involved no collection or manipulation of physical or biological samples, institutional ethics committee approval was not required. Field surveys were conducted in public access areas, were entirely non-invasive, and did not disturb any endangered or protected species.

At each site, benthic data were collected along six 10 m transects in two parallel sets of three. Transect sets were 5 m apart and all sets were oriented parallel to shore. Surface distance (in cm) for all benthic components directly under each transect were measured using a flexible measuring tape. The following benthic components were recorded and measured: scleractinian hard corals, crustose coralline algae (CCA), macroalgae, turf algae, sand, rubble, carbonate hard substrate, non-carbonate hard substrate, or other (including cyanobacteria, soft coral, seagrass, sponges, zoanthids, corallimorphs, or other invertebrates). Corals were identified to species-morphology or genus-morphology taxonomic level (S1 Table) following NCRMP protocols.

For urchins, species abundance and test-size class data were collected for the following bioeroding urchin genera commonly found in Hawaiʻi: *Echinometra, Echinothrix, Diadema,* and *Echinostrephus.* Urchin surveys were conducted within a 1 m width by 10 m length belt along the six benthic transects. Test size of each urchin was measured using a ruler and binned into 20 mm size classes from 0–160 mm.

For parrotfish, data were collected for the following parrotfish genera commonly found in Hawaiʻi: *Calotomus, Chlorurus,* and *Scarus*. Parrotfish species, life phase, abundance, and total length were recorded along ten replicate 5 m width by 30 m length belt transects running parallel (n = 6) and perpendicular (n = 4) to the reef crest, and separated by 5 m. Total fish length was estimated visually and binned into the following 10 cm size classes from 0–60 cm.

### Gross carbonate production

Coral and CCA carbonate production for each observed individual colony was calculated by incorporating surface distance (cm) and literature-derived regional species- or genera-specific linear extension rates (cm yr^-1^) and skeletal density (g cm^-3^) into morphology-specific production equations [20]. Gross carbonate production (kg CaCO_3_ m^-2^ yr^-1^) was calculated as the total sum of carbonate production by coral and CCA and summed per taxa and across all taxa for each transect. Transects were then averaged to calculate site-level mean gross carbonate production rates.

#### Calcification rates databases.

Coral and CCA carbonate production rates were calculated using two calcification rates databases: (1) a genus-level, Indo-Pacific database [20] containing coral linear extension rates and skeletal density from across the Indo-Pacific, and (2) a species- and genus-level, Pacific Islands database that includes regionally constrained linear extension rates and skeletal density at the species-level (when available) and genus-level. The two databases of coral and CCA linear extension (cm yr^-1^), skeletal density (g cm^-3^), and calcification rates (g cm^-2^ yr^-1^) were compared based on their taxonomic resolution (genus or species-level) and geographic range. Both databases include *in situ* measurements and control-only experimental studies, and the methods used to measure coral growth rates are described within the respective databases. Studies reporting growth rates from corals exposed to experimental treatments were excluded. Details of each database are provided below.

1. Genus-level, Indo-Pacific: existing *ReefBudget* Indo-Pacific calcification rates database (see v1.3 [20] for detailed information of coral growth rate studies included in this database). Database includes coral linear extension rates and skeletal density data from studies conducted throughout the wider tropical Indian and Pacific Oceans, averaged for each genus and morphology group (e.g., massive *Porites*).
2. Species/genus-level, Pacific-Islands: updated version of the *ReefBudget* Indo-Pacific calcification rates database [20] for the Pacific Islands region. Coral growth rate data were restricted to studies conducted within the Pacific Islands spatial domain (125°E to 155°W, 28°N to 28°S; Fig 1B). This latitudinal range reflects the generally accepted global limits of coral reef occurrence, while the longitudinal bounds were selected to include Oʻahu and surrounding islands and to ensure sufficient study coverage across the central, southern and western Pacific Ocean regions. Although studies solely from within the relatively higher latitudes of the Hawaiian Archipelago would most accurately represent coral growth rates on Oʻahu, the limited availability of such studies necessitated inclusion of data from the broader Pacific Islands region. Following the taxonomic levels used by NCRMP (see SI in [53] for the full list of included studies), coral growth rates were averaged at the species-level when possible. Corals that could not be identified to species were averaged at the genus-level. CCA calcification rates were estimated from Calcification Accretion Units (CAUs) collected and analyzed as part of NCRMP monitoring missions [54].

#### Benthic metrics.

Percent cover for each benthic component (e.g., scleractinian hard coral) was calculated as the sum of surface cover of the benthic component divided by the total transect cover of all benthic components. Coral diversity (H’) was calculated for each transect using the Shannon diversity index method [55,56]. Rugosity for each transect was calculated by dividing the total sum of the surface cover of all observed benthic components on the transect by the linear length (10 m). Site-level metrics were then calculated as the average of the six transects per site.

### Carbonate bioerosion

Calcium carbonate erosion by urchins, parrotfish, macroborers, and microborers were individually calculated per transect and averaged for each site, and then summed for total carbonate bioerosion (kg CaCO_3_ m^-2^ yr^-1^) per site [20]. Urchin erosion rates were calculated for each genus and size class as a function of test size, summed across all observed urchin genera, and divided by transect surface area to estimate transect-level urchin erosion. Erosion rates of individual parrotfishes were calculated using a two-step approach. First, foraging metrics, including bite rate, bite volume, and the proportion of bites that leave scars, were calculated as functions of the individual’s species, life phase, and total length [57]. Second, foraging metrics were used to estimate erosion rates for each parrotfish species and size class. Erosion rates were then summed across all species and divided by surface area to estimate transect-level parrotfish erosion. Parrotfish biomass was calculated using length-to-weight conversion parameters from the FishBase database [58,59]. Macrobioerosion (e.g., sponges, bivalves, worms) and microbioerosion (e.g., cyanobacteria, chlorophytes, fungi) rates were calculated from transect rugosity, percent cover of erodible carbonate substrate (total cover of macroalgae, turf algae, pavement, and rubble), and literature-derived Indo-Pacific bioerosion rates following *ReefBudget* methodology [20].

### Net carbonate production

Site-level estimates of net carbonate production (kg CaCO_3_ m^-2^ yr^-1^) were calculated as gross carbonate production minus the total carbonate bioerosion [16]. Gross carbonate production estimates derived from the ‘Species/genus-level, Pacific Islands’ database were used in net production calculations.

### Data analysis

All data analyses and statistical tests were performed in R version 4.3.1. Carbonate production and erosion estimates were calculated using R processing scripts derived from the Indo-Pacific *ReefBudget* datasheets [20]. Spatial visualizations, including maps of research sites, were generated in R using the ‘ggplot2’ and ‘mapdata’ packages, with basemaps derived from the open-source world2Hires dataset (GNU General Public License v2). Paired sample *t*-tests were conducted to compare coral linear extension rates and skeletal density between the two calcification rate databases ('Genus-level, Indo-Pacific' and 'Species/genus-level, Pacific Islands') using matched genera-morphology taxa represented in both databases and recorded at the Oʻahu sites. One-way and two-way analyses of variance (ANOVA) were used to evaluate differences among sites and between calcification rate databases. A two-way ANOVA, followed by post hoc Tukey tests on square-root transformed data, was used to evaluate the effect of database (‘Genus-level, Indo-Pacific’ and ‘Species/genus-level, Pacific Islands’), site, and their interaction on gross carbonate production. Linear and multiple regression analyses were used to assess relationships among gross carbonate production, total carbonate bioerosion, and net carbonate production, as well as benthic metrics (percent coral cover, coral genera diversity, and reef rugosity) and bioeroder metrics (urchin density, urchin mean test size, parrotfish biomass and abundance). Additionally, an analysis of covariance (ANCOVA; linear model with interaction terms) was used to test whether the relationship between coral cover and gross carbonate production differed among coral genus-morphology groups. Following Perry et al. [17], a confidence rating (High, Medium, or Low) was assigned to each carbonate budget component based on the level of confidence in the survey methods and supporting datasets used (see Table 3 in Perry et al. [17]).

## Results

### Gross carbonate production and benthic metrics

The two calcification rate databases ('Genus-level, Indo-Pacific' and 'Species/genus-level, Pacific Islands') did not differ significantly when comparing coral linear extension rates (*t*(10) = −0.53, *p* = 0.607; S1 Fig) or skeletal density (*t*(10) = −0.01, *p* = 0.99) for coral taxa (genus-morphology) observed across Oʻahu sites.

Gross carbonate production estimates also did not differ when calculated using the two calcification rates databases (two-way ANOVA, *F*_*(1,70)*_ *= 3.422, p* = 0.069); however, gross carbonate production did vary significantly across sites (*F*_*(6,70)*_ *= 37.58, p* < 0.001; Fig 1C), with site means (± SE) ranging from 0.52 ± 0.18 (ʻEwa) to 7.10 ± 0.81 kg CaCO_3_ m^-2^ yr^-1^ (Kāneʻohe Bay) (Table 1). Kāneʻohe Bay and Reef Runway exhibited significantly higher gross carbonate production than all other sites (Bonferroni corrected t-test, *p* < 0.001), while the remaining sites were similar (Fig 1C). There was no interaction between database selection and site (*F*_*(6,70)*_ *= 0.05, p =* 0.99). Gross carbonate production was largely driven by coral carbonate production, as corals were the dominant carbonate producer across all sites (range: 0.52 ± 0.18 to 7.08 ± 0.81 kg CaCO_3_ m^-2^ yr^-1^; Table 1) and contributed over 98% to total gross carbonate production. CCA carbonate production was minor to negligible, ranging from 0.003 ± 0.002 (ʻEwa) to 0.06 ± 0.03 (Barbers Point) kg CaCO_3_ m^-2^ yr^-1^ (Table 1).

**Table 1:** Summary of site characteristics and individual biologically derived carbonate production and erosion rates in kg m^-2^ yr^-1^ (± SE).

Site								
	CR	Mākua	Mokulēʻia	Kāneʻohe Bay	Kewalo	Reef Runway	ʻEwa	Barbers Point
Site depth (m)		14.6	14	12.5	12.2	9.5	10.3	11
Site location (latitude °N, longitude °E)		21.5341, −158.2344	21.59095, −158.17467	21.4798, −157.7830	21.2884, −157.8653	21.2981, −157.9330	21.2934, −158.0060	21.2903, −158.0670
Substrate rugosity		1.13 ± 0.03	1.07 ± 0.03	1.28 ± 0.07	1.12 ± 0.02	1.43 ± 0.05	1.13 ± 0.04	1.34 ± 0.02
Hard coral cover (%)		3.89 ± 1.76	9.51 ± 2.42	59.4 ± 2.22	14.58 ± 5.34	58.39 ± 7.68	3.69 ± 1.22	10.6 ± 1.56
CCA cover (%)		1.28 ± 0.87	0.76 ± 0.63	2.87 ± 1.17	1.25 ± 0.88	7.4 ± 3.61	0.53 ± 0.31	3.91 ± 1.43
Coral genera diversity (H’)		0.49 ± 0.16	1.33 ± 0.08	1.70 ± 0.09	1.00 ± 0.23	1.38 ± 0.10	0.71 ± 0.17	1.13 ± 0.12
**Gross carbonate production (species/genus-level, Pacific Islands)**		**0.66 ± 0.27**	**1.09 ± 0.18**	**7.10 ± 0.81**	**1.92 ± 0.71**	**4.83 ± 0.63**	**0.52 ± 0.19**	**1.49 ± 0.32**
Gross carbonate production (genus-level, Indo-Pacific)		0.81 ± 0.33	1.59 ± 0.30	8.33 ± 0.91	2.30 ± 0.84	6.01 ± 0.87	0.73 ± 0.26	1.96 ± 0.37
Coral production	H/M	0.66 ± 0.27	1.08 ± 0.18	7.08 ± 0.81	1.91 ± 0.71	4.77 ± 0.62	0.52 ± 0.18	1.46 ± 0.32
Dominant coral genera total percent cover (%) and percent contribution to site gross carbonate production (%)		Massive *Porites* (cover: 2.68 prod: 85.15)	Branching *Pocillopora* (cover: 5.43 prod: 46.0)	Encrusting *Montipora* (cover: 23.32 prod: 22.53)	Massive *Porites* (cover: 10.15 prod: 80.39)	Encrusting *Montipora* (cover: 39.36 prod: 39.86)	Branching *Pocillopora* (cover: 1.70 prod: 36.46)	Branching *Pocillopora* (cover: 5.45 prod: 52.24)
		Branching *Pocillopora* (cover: 0.48 prod: 8.61)	Massive *Porites* (cover: 1.70 prod: 28.63)	Massive *Porites* (cover: 21.42 prod: 55.84)	Encrusting *Montipora* (cover: 3.46 prod: 14.30)	Branching *Pocillopora* (cover: 8.40 prod: 24.87)	Massive *Porites* (cover: 1.68 prod: 56.67)	Massive *Porites* (cover: 2.87 prod: 36.36)
		Encrusting *Montipora* (cover: 0.03 prod: 1.40)	Encrusting *Montipora* (cover: 1.20 prod: 24.17)	Branching *Pocillopora* (cover: 6.52 prod: 10.54)	Branching *Pocillopora* (cover: 0.80 prod: 4.27)	Massive *Porites* (cover: 7.50 prod: 30.72)	Encrusting *Montipora* (cover: 0.10 prod: 6.20)	Encrusting *Montipora* (cover: 1.95 prod: 8.85)
CCA production	H/M	0.008 ± 0.005	0.004 ± 0.004	0.02 ± 0.01	0.008 ± 0.005	0.06 ± 0.03	0.003 ± 0.002	0.03 ± 0.01
**Total bioerosion**		**0.34 ± 0.06**	**0.46 ± 0.04**	**0.76 ± 0.10**	**0.80 ± 0.15**	**1.45 ± 0.20**	**0.67 ± 0.17**	**1.15 ± 0.10**
Urchin erosion	H/H	0.05 ± 0.02	0.03 ± 0.004	0.43 ± 0.05	0.46 ± 0.12	1.05 ± 0.09	0.28 ± 0.12	0.60 ± 0.09
Dominant urchin spp. (indiv. site^-1^)		*Echinostrephus sp.* (2)	*Echinometra sp.* (10)	*Echinometra sp.* (60)	*Echinothrix sp.* (15)	*Echinothrix sp.* (17)	*Echinothrix sp.* (3)	*Echinometra sp.* (75)
Parrotfish erosion	M/H	0	0.003 ± 0.003	0.10 ± 0.04	0.02 ± 0.02	0.17 ± 0.06	0.001 ± 0.001	0.02 ± 0.01
Dominant parrotfish spp. (indiv. site^-1^)		No parrotfish (0)	*C. carolinus* (2)	*C. spilurus* (70)	*S. psittacus* (13)	*C. spilurus* (24)	*C. carolinus* (7)	*S. rubroviolaceus* (17)
Macrobioerosion	L/L	0.13 ± 0.02	0.19 ± 0.01	0.10 ± 0.01	0.15 ± 0.01	0.12 ± 0.02	0.18 ± 0.02	0.24 ± 0.003
Microbioerosion	L/L	0.16 ± 0.02	0.24 ± 0.02	0.12 ± 0.01	0.18 ± 0.01	0.12 ± 0.03	0.21 ± 0.07	0.28 ± 0.005
**Net biological carbonate production**		**0.33 ± 0.27**	**0.62 ± 0.18**	**6.34 ± 0.82**	**1.12 ± 0.77**	**3.38 ± 0.67**	**−0.15 ± 0.34**	**0.34 ± 0.38**

Reported metrics and confidence ratings (CR) are based on the approach from Perry et al. [17] and modified for this study. CR is reported below as the level (High, Medium, Low) of confidence for survey method/confidence in supporting datasets.

**Fig 2:**
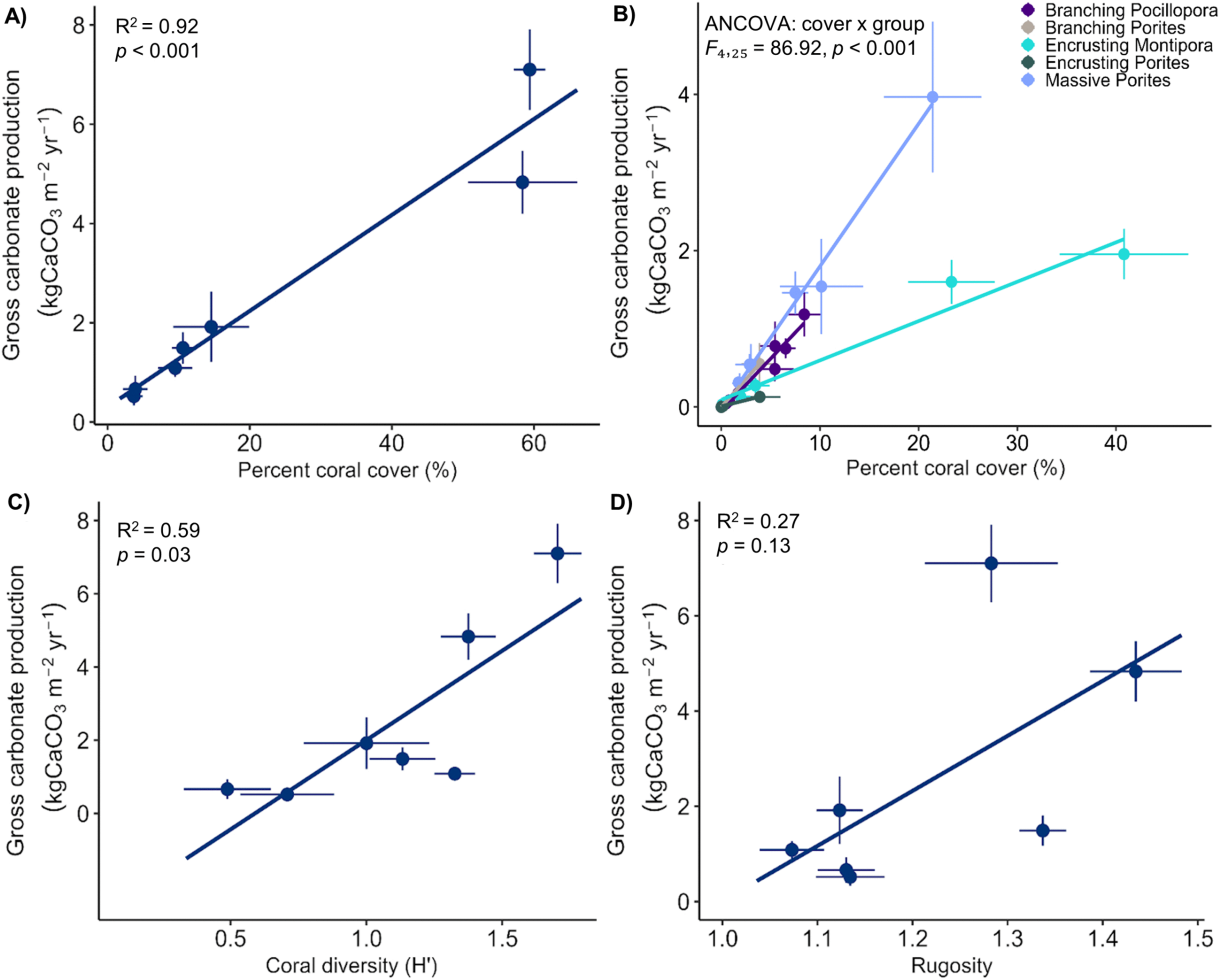
Scatter plots showing relationships between gross carbonate production and (A) site-level mean ± standard error (SE) percent coral cover, (B) mean ± SE percent coral cover for common Hawaiʻi coral genera surveyed, (C) site-level mean ± SE coral genera diversity, and (D) site-level mean ± SE rugosity. All gross carbonate production data were calculated using the ‘Species/genus-level, Pacific Islands’ database.

Coral cover (%) varied across sites (one-way ANOVA, *F*_*(6,35)*_ *= 41.22*, *p* < 0.001) and was a strong predictor of gross carbonate production (b = 0.13, R^2^ = 0.92, *p* < 0.001; Fig 2A). Hard coral cover was highest at Kāneʻohe Bay (59.40 ± 2.22%) and Reef Runway (58.39 ± 7.68%), where gross carbonate production estimates were also higher than all other sites (Table 1). Conversely, hard coral cover was lowest at sites ʻEwa (3.69 ± 1.22%) and Mākua (3.89 ± 1.76%), which also had the lowest gross carbonate production (Table 1). The relationship between coral cover and gross carbonate production was positive for all coral taxa; however, the interaction between coral cover and genus-morphology group was significant (ANCOVA, *F*_*(4,25)*_ = *86.92*, *p <* 0.001), indicating that the slope of the coral cover-production relationship differed among genus-morphology groups. Relative to the overall relationship, encrusting *Montipora* (b = −0.08, *p <* 0.001) and encrusting *Porites* (b = −0.10, *p* = 0.009) exhibited weaker increases in carbonate production with increasing cover, whereas massive *Porites* (b = 0.05, *p* = 0.007) exhibited a stronger increase (Fig 2B).

Coral genera diversity (H’) showed significant variation among sites (one-way ANOVA, *F*_*(6,35)*_ *= 8.17*, *p* < 0.001) and was a predictor for gross carbonate production (b = 4.88, R^2^ = 0.59, *p* = 0.03; Fig 2C). Thirteen species across five genera were observed across sites. Highest diversity was at Kāneʻohe Bay where coral percent cover and gross carbonate production were also highest, while Mākua was the least diverse site (Table 1). When summed across all sites, massive *Porites,* encrusting *Montipora*, and branching *Pocillopora* were the three dominant coral-morphology taxa contributing to carbonate production and percent coral cover (Table 1 and S2 Fig), accounting for ~94% of the gross carbonate production and ~93% of total percent coral cover. Massive *Porites* contributed most to gross carbonate production across sites (49.63 ± 6.92%) and was second highest in total coral cover. Encrusting *Montipora* had the highest total coral cover and contributed 24.38 ± 2.78% to gross carbonate production. Branching *Pocillopora* was the third highest contributor to coral cover and gross carbonate production, accounting for 20.33 ± 2.83% of gross carbonate production.

Site-level differences in reef rugosity were also observed (one-way ANOVA, *F*_*(6,35)*_ *= 10.92*, *p* < 0.001), with highest rugosity at Reef Runway and lowest at Mokulēʻia (Table 1). Although rugosity was positively associated with coral cover and gross carbonate production, neither relationship was statistically significant (coral cover: b = 132.71, R^2^ = 0.42*, p* = 0.07; gross carbonate production: b = 11.55, R^2^ = 0.27*, p* = 0.13; Fig 2D).

### Carbonate bioerosion

**Fig 3:**
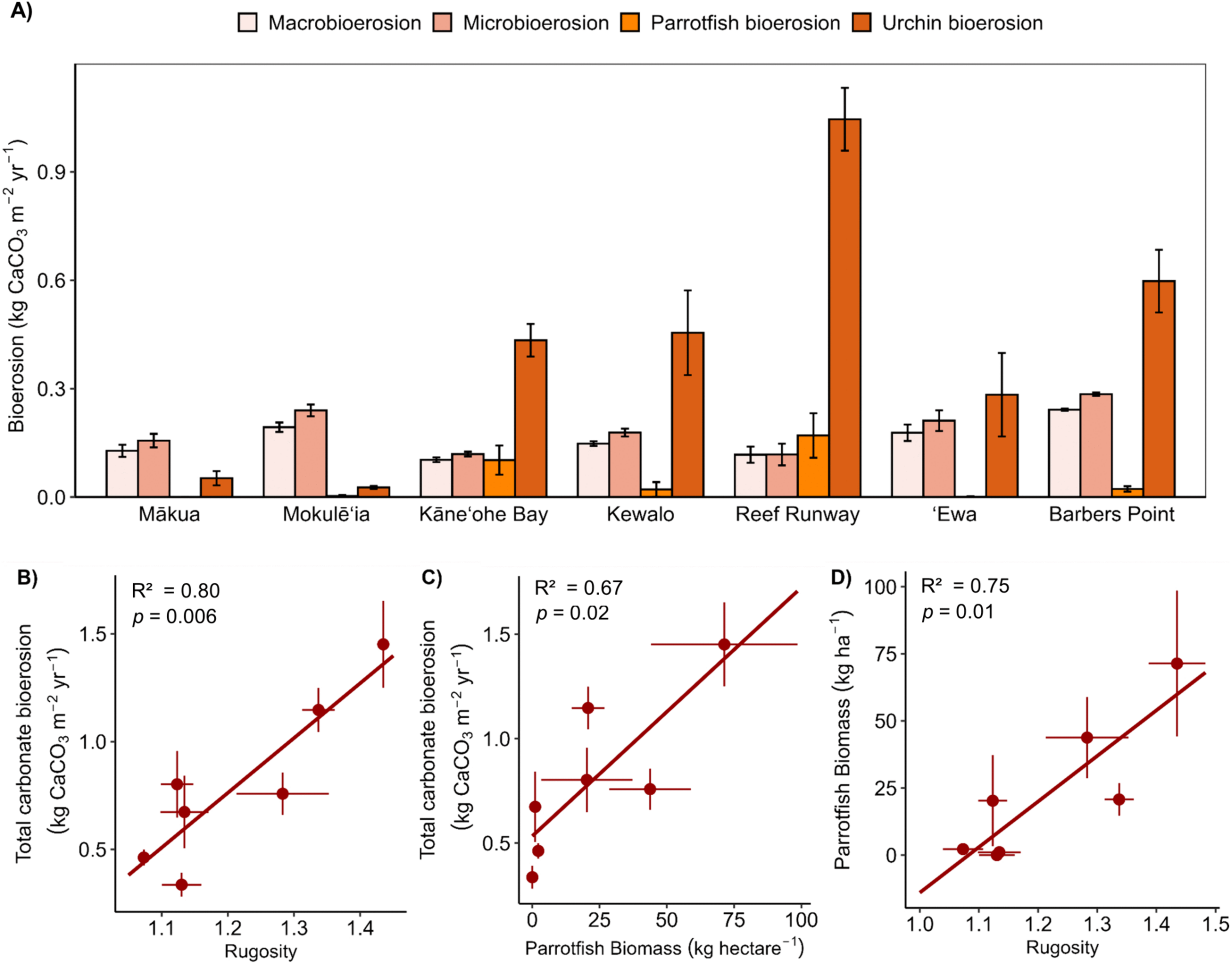
(A) Contributions of major biological eroder groups (mean ± SE) to total carbonate bioerosion across seven Oʻahu survey sites. (B-D) Scatter plots showing relationships among site-level mean ± SE total carbonate bioerosion, reef rugosity, and parrotfish biomass.

Total carbonate bioerosion from urchins, parrotfish, macroborers, and microborers varied across sites (one-way ANOVA, *F*_*(6,35)*_ *= 11.01*, *p* < 0.001), ranging from 0.34 ± 0.06 at Mākua to 1.45 ± 0.20 kg CaCO_3_ m^-2^ yr^-1^ at Reef Runway (mean ± SE), but was less variable than gross carbonate production (Table 1). Urchin erosion (range: 0.03 ± 0.004 to 1.05 ± 0.09 kg CaCO_3_ m^-2^ yr^-1^) was the largest contributor to total carbonate bioerosion at five sites (Kāneʻohe Bay, Kewalo, Reef Runway, ʻEwa, and Barbers Point), whereas microbioerosion (range: 0.12 ± 0.01 to 0.28 ± 0.005 kg CaCO_3_ m^-2^ yr^-1^) was highest at Mākua and Mokulēʻia (Fig 3A). Urchin density was highest at Barbers Point (1.63 ± 0.07 ind. m^-2^) and Kāneʻohe Bay (1.27 ± 0.10 ind. m^-2^). Parrotfish erosion (range: 0 to 0.17 ± 0.06 kg CaCO_3_ m^-2^ yr^-1^) and macrobioerosion (range: 0.10 ± 0.01 to 0.24 ± 0.003 kg CaCO_3_ m^-2^ yr^-1^) were comparatively minor contributors at all sites (Table 1, Fig 3A).

Rugosity was a strong predictor of urchin erosion (b = 2.32, R^2^ = 0.80, *p* = 0.006), the primary contributor to total carbonate bioerosion, resulting in a similarly strong positive relationship between rugosity and total carbonate bioerosion (b = 2.54, R^2^ = 0.80, *p* = 0.006; Fig 3B). Interestingly, while rugosity showed a positive trend with urchin density, it did not significantly predict urchin density (b = 2.68, R^2^ = 0.33, *p* = 0.18) or mean urchin test size (b = −17.11, R^2^ = 0.02, *p* = 0.78) at the site level. Coral cover was also not a significant predictor of urchin density (b = 0.52, R^2^ = 0.11, *p* = 0.46), mean urchin test size (b = −0.11, R^2^ = 0.02, *p* = 0.74), or urchin erosion (b = 0.009, R² = 0.45, *p* = 0.10). A multiple linear regression indicated that total bioerosion was not significantly related to both urchin density and mean urchin test size (R² = 0.44, Adjusted R² = 0.16, *F₂,₄ = 1.57*, *p* = 0.31). Urchin density (b = 0.0085, *p* = 0.16) and mean test size (b = 0.0137, *p* = 0.26) were both positively associated with total bioerosion, although neither were statistically significant.

Parrotfish erosion was positively related to rugosity (b = 0.40, R^2^ = 0.69, *p* = 0.02) and percent coral cover (b = 0.002, R^2^ = 0.90, *p* = 0.001). Parrotfish biomass, which incorporates both the number of individuals and their sizes, was positively related to total carbonate bioerosion (b = 56.47, R^2^ = 0.67, *p =* 0.02; Fig 3C), rugosity (b = 169.91, R^2^ = 0.75, *p* = 0.01; Fig 3D), and coral cover (b = 0.98, R^2^ = 0.85, *p* = 0.003). In contrast, parrotfish abundance alone was positively associated with coral cover (b = 10.21, R^2^ = 0.55, *p* = 0.05), but was not significantly associated with total bioerosion (b = 0.0002, R^2^ = 0.04, *p* = 0.67) or rugosity (b = 1043.00, R^2^ = 0.17, *p* = 0.36), indicating that body size, rather than abundance alone, better explained variation in parrotfish-driven bioerosion.

Rugosity was not significantly associated with macrobioerosion (b = −0.05, R^2^ = 0.02, *p* = 0.76) or microbioerosion (b = −0.13, R^2^ = 0.08, *p* = 0.55). Macrobioerosion and microbioerosion both showed negative relationships with coral cover, although these were not statistically significant at the site level (macrobioerosion: R^2^ = 0.41, *p* = 0.12; microbioerosion: R^2^ = 0.51, *p* = 0.07).

### Net carbonate production

**Fig 4:**
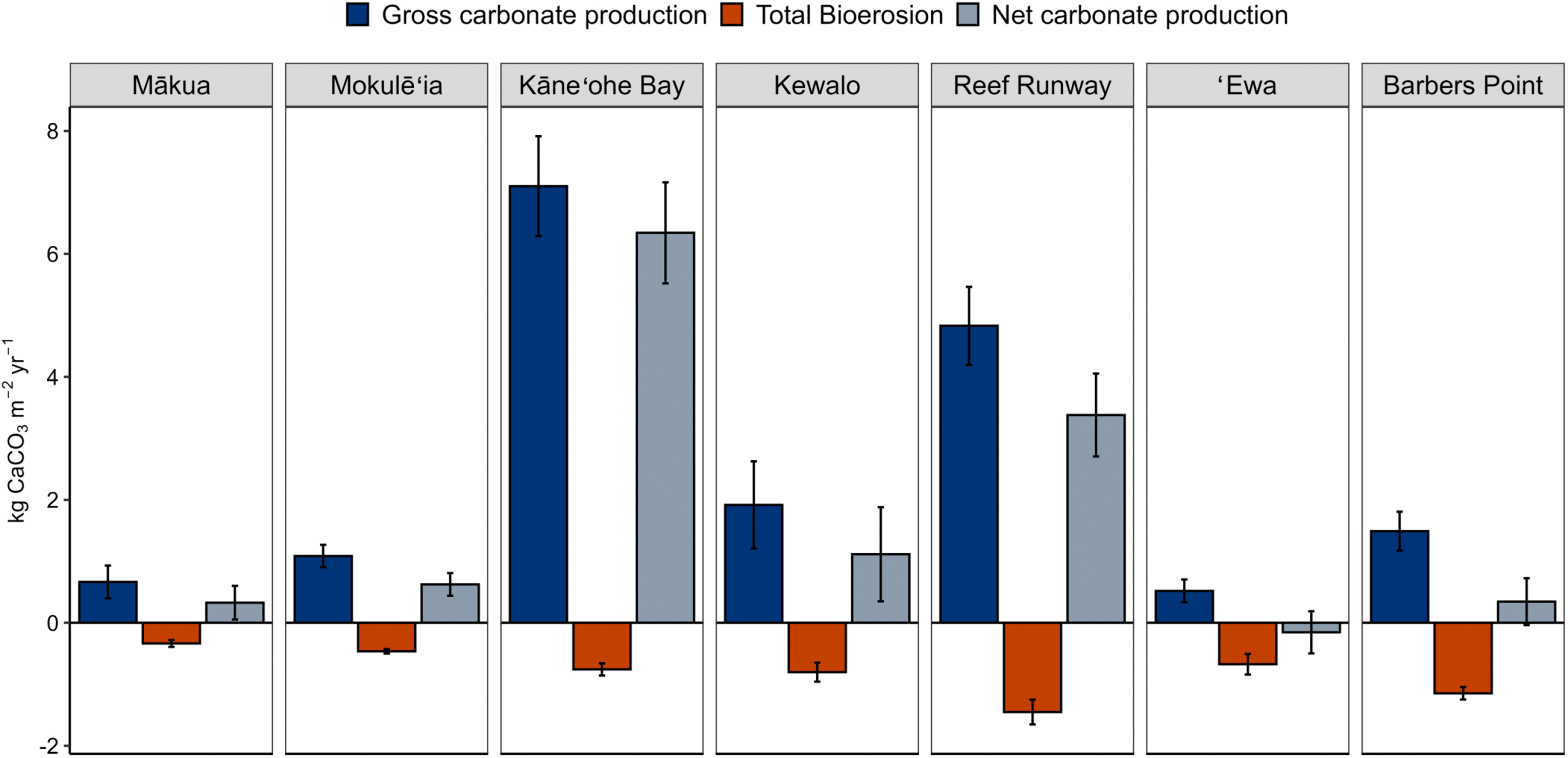
Gross carbonate production, total carbonate bioerosion, and net carbonate production rates (mean ± SE) estimated for seven Oʻahu survey sites. Total carbonate bioerosion rates are represented here as negative (-) kg CaCO_3_ m^-2^ yr^-1^ for the visualization of removal of carbonate material by bioeroders.

Net carbonate production estimates ranged from −0.15 ± 0.34 kg CaCO_3_ m^-2^ yr^-1^ at ‘Ewa to 6.34 ± 0.82 kg CaCO_3_ m^-2^ yr^-1^ at Kāneʻohe Bay (mean ± SE). Highest net carbonate production occurred at Kāneʻohe Bay and Reef Runway, with the lowest net carbonate production at ‘Ewa, Mākua, and Barbers Point (Table 1, Fig 4). Gross carbonate production was a strong predictor for net carbonate production (b = 0.93, R^2^ = 0.97, *p* < 0.001), whereas no significant relationship was found between total carbonate bioerosion and net carbonate production (b = −1.87, R^2^ = 0.09, *p* = 0.50).

## Discussion

### Net carbonate production on Oʻahu reefs

Across sites, six of the seven reefs exhibited net positive carbonate production, although Mākua (0.33 ± 0.27 kg CaCO₃ m ⁻² yr ⁻¹) and Barbers Point (0.34 ± 0.38 kg CaCO₃ m ⁻² yr ⁻¹) were near the threshold required to sustain framework growth. ʻEwa was the only site with slightly negative net carbonate production (−0.15 ± 0.34 kg CaCO₃ m ⁻² yr ⁻¹), with both ʻEwa and Barbers Point estimates within error of zero, indicating functionally neutral budget states. Despite Oʻahu’s dense human population, these results suggest that the majority of these reefs are still maintaining positive framework growth. The strong spatial variability observed in coral cover—and its close association with gross and net carbonate production—across Oʻahu sites aligns with coral species distribution models and prior studies that identify wave energy (e.g., mean and maximum significant wave height) as a primary driver of coral distribution throughout Oʻahu and the Hawaiian Archipelago [60–65]. Northwest sites Mākua and Mokulēʻia had low coral cover, rugosity, and carbonate production, consistent with their susceptibility to large winter north and northwestern swells, with Mākua additionally exposed to south-southwest summer swell due to its position along the coastline. In contrast, Kāneʻohe Bay supported the highest net carbonate production rates and high coral cover, likely associated with its limited wave exposure [35,50]. South coast sites (Kewalo, Reef Runway, ʻEwa, and Barbers Point), while similarly subject to summer southerly swell, varied substantially in net carbonate production, suggesting that site specific differences in coastal orientation and anthropogenic influence may contribute to variability in coral cover and reef growth dynamics. For example, Reef Runway exhibited relatively high coral cover and net carbonate production, potentially reflecting reduced human access and physical disturbance associated with its proximity to airport and military infrastructure, whereas ʻEwa reef, adjacent to the urbanized and industrialized ʻEwa region, had lower coral cover and slightly negative carbonate budgets (Table 1). Overall, spatial variability in reef carbonate budgets across Oʻahu appears strongly linked to coral cover patterns likely driven by a combination of geomorphic setting and localized anthropogenic pressures (e.g., land-use).

While census-based carbonate budget assessments for Oʻahu reefs remain limited, our net carbonate production estimates align well with the ranges reported using various methodologies. Most prior work has focused on the windward side of Oʻahu, including census-based estimates for Kailua Bay (0.3 kg m^-2^ yr^-1^, [66]), and hydrochemical estimates for Kāneʻohe Bay (6.3 to 12.2 kg m^-2^ yr^-1^ [67], 0.5 to 0.9 kg m^-2^ yr^-1^ [41]) and Waimānalo (4.5 to 5.8 kg m^-2^ yr^-1^ [68]). These values vary due to methodological differences and spatial and temporal variability in calcification rates and reef community composition; however, all net production estimates—including our estimate for Kāneʻohe Bay (6.34 ± 0.82 kg m^-2^ yr^-1^)—indicate a positive carbonate production state for this windward reef system. In 2015, carbonate blocks were deployed at a subset of our sites around Oʻahu, and net accretion was estimated from percent changes in carbonate block volume [30]. This approach quantifies net changes in carbonate substrate rather than biogenic carbonate production and erosion, but similar spatial trends were observed, with highest net accretion in Kāneʻohe Bay and lowest at Mākua. Although these methods quantify different components of reef carbonate dynamics and are not directly comparable in magnitude, together they provide a useful basis for evaluating spatial patterns in carbonate production and erosion dynamics across Oʻahu reefs. More recently, net carbonate production estimates derived using similar census-based methods and Structure-from-Motion photogrammetry at the same Oʻahu sites ranged from −0.1 to 7.8 kg CaCO_3_ m^-2^ yr^-1^ [69], closely spanning the range we observed in this present study.

Across the U.S. Pacific Islands region, the average net carbonate production estimated from Oʻahu sites in this study (1.7 ± 0.9 kg m^-2^ yr^-1^) was near the regional mean reported for coral reef sites in the Mariana Islands, Northwestern Hawaiian Islands, Pacific Remote Islands, and American Samoa (2.1 ± 0.6 kg m^-2^ yr^-1^ [34]). Oʻahu estimates were less than half the average from broader Pacific carbonate budget assessments (3.7 ± 0.4 kg m^-2^ yr^-1^ [21]), except for Reef Runway and (3.4 ± 0.7) and Kāneʻohe Bay (6.3 ± 0.8 kg m^-2^ yr^-1^), which approached or exceeded the regional mean. Previous studies have shown that coral growth and calcification can vary substantially across taxa and environmental conditions [70], and that Hawaiian reefs, as relatively high-latitude reef systems, tend to exhibit slower coral calcification and reef accretion than many tropical Pacific reefs [71–74]. These generally lower coral and CCA calcification rates, together with the synergistic effects of high anthropogenic pressure and recent bleaching events (2014, 2015 [42], 2019 [75]), can likely contextualize the relatively slower reef framework production observed across Oʻahu reefs. Given that nearly half of the Oʻahu sites exhibited relatively low rates of reef framework production, continued monitoring is increasingly important as environmental perturbations are expected to intensify.

### Influence of database on gross carbonate production and database limitations

Coral calcification rates averaged within the Pacific Islands region did not significantly differ from those averaged across the broader Indo-Pacific (S1 Fig), resulting in comparable estimates of gross carbonate production between approaches. Previous studies have reported reduced coral growth rates in the relatively higher-latitude Hawaiian Archipelago compared to many equatorial and subequatorial reefs in the Indo-Pacific [70–74], suggesting that the overall effect size observed in the present study may be underestimated because growth rates were derived from Pacific Islands-wide datasets rather than exclusively from the Hawaiian Archipelago. This underscores the importance of refining growth rate inputs to reflect regional and local environmental conditions, as locally derived coral growth rates can substantially influence carbonate production estimates where environmental conditions enhance or reduce coral calcification [45,48]. However, the ability to implement geographically and taxonomically refined coral calcification databases remains limited by data availability. Overall, we found that coral growth rate studies conducted specifically within the Pacific Islands region were relatively sparse (Pacific Islands = 23 studies), compared to the broader Indo-Pacific database (Indo-Pacific = 121 studies, including Pacific Islands studies). Additionally, species-specific growth rates were also limited for many of the coral taxa commonly found on Oʻahu, with available studies conducted spanning from years 1924–2021. Future investment in region-specific and species-specific coral linear extension and skeletal density studies would continue to improve the representativeness of carbonate budget assessments in Hawaiʻi and other Pacific Island reef systems with high species diversity and variability in oceanographic conditions [21]. Filling these data gaps would allow coral growth rate inputs underpinning gross carbonate production calculations to better reflect local environmental conditions [76] and biogeographies that influence reef habitat growth. Although this study represents an effort to refine coral growth rate inputs for Oʻahu, currently available values remain derived at the broader Pacific Islands regional scale rather than at the island (Oʻahu) or archipelago (Hawaiian Archipelago) scale. Continued development of regionally and locally-relevant calcification rates databases, alongside increased availability of site-level data, may improve confidence in future carbonate budget estimates.

### Gross carbonate production and relationships between coral metrics

While total coral cover and gross production were tightly linked, our results also highlight the disproportionate influence of several coral taxa on site-level framework production. For example, at the site with the highest carbonate production and coral cover (Kāneʻohe Bay), both encrusting *Montipora* and massive *Porites* were most abundant with similar cover. Yet, *Porites* contributed approximately twice as much to carbonate production than *Montipora* (S3 Fig). At Reef Runway, the second-highest site for carbonate production and coral cover, encrusting *Montipora* had approximately four times the cover of massive *Porites*, yet both taxa contributed equally (~20%) to carbonate production (S3 Fig). This result is due to differences in average colony-level production rates between massive *Porites* (mean ± SE = 25.7 ± 1.3 g CaCO_3_ yr^-1^) and encrusting *Montipora* (mean ± SE = 7.5 ± 0.1 g CaCO_3_ yr^-1^), which is a function of linear extension rate, skeletal density, and colony size. Previous studies have shown that reefs with similar total coral cover but different coral composition (i.e., slow-growing versus fast-growing corals) could exhibit markedly different estimates of gross carbonate production, emphasizing the importance of species identity over total coral cover alone. For example, in the Caribbean and Florida, disturbance- and disease-driven shifts toward stress-tolerant, slow-growing coral taxa substantially reduced reef-wide calcification capacity, net carbonate production, and structural complexity, even with minimal changes in total coral cover [77–82]. While our study found coral cover was the primary driver of gross and net carbonate production on Oʻahu, site-specific genus-morphology assessments are still critical to understand underlying mechanisms of carbonate budgets on a global scale.

Across Oʻahu, coral assemblages were dominated by massive *Porites*, encrusting *Montipora*, and branching *Pocillopora*, consistent with species distribution models for the Main Hawaiian Islands, which identify *P. meandrina*, *M. capitata*, and *P. lobata* as three of the most abundant reef-building corals across islands [60]. Long-term monitoring across Hawaiʻi also indicates relatively limited shifts in dominant coral taxa over time [62,64,83], suggesting that future changes in gross carbonate production across Oʻahu may be more strongly driven by reductions in live coral cover than by shifts in coral community composition. Yet, even subtle shifts in the relative dominance of these coral taxa could disproportionately influence reef carbonate budget trajectories. Notably, these taxa have been found to differ in their susceptibility to environmental stressors. Massive *Porites* is comparatively more tolerant of thermal stress and sedimentation than branching taxa such as *P. meandrina* [84–86], while encrusting *Montipora* (e.g., *M. capitata*) can persist across habitats with variable environmental and physical conditions [87]. Consequently, future increases in anthropogenic and environmental stress—such as more frequent and intense marine heatwaves, or the introduction of coral diseases—may alter the relative contributions of these dominant framework-building taxa, with important implications for long-term net carbonate production and structural maintenance.

Higher coral diversity often includes multiple key framework-building species, supporting a positive relationship with carbonate production; however, reefs with low diversity can still sustain high production rates depending on the traits of the dominant taxa [34]. At our Oʻahu sites, these three genera–morphology taxa (massive *Porites*, encrusting *Montipora*, branching *Pocillopora*) played a central role in maintaining reef structure and contributing to carbonate production despite relatively low overall species diversity. These findings highlight that while greater species diversity can support reef function and resilience [88,89], it does not always correlate with highest carbonate production estimates, particularly when key reef-building species are absent. Distinguishing which coral species are maintaining the reef and quantifying their relative contributions to gross carbonate production can minimize misinterpretations of the generalized linear relationships between coral cover, reef rugosity, coral diversity, and gross carbonate production. These dynamics have important implications for reef restoration. Supporting reef health and persistence may require balancing fast-growing, carbonate-producing species with those that offer greater resilience to environmental stress and functional redundancy to sustain ecosystem processes.

Structural complexity, as quantified by rugosity, was generally a poor predictor for gross carbonate production on Oʻahu. Reef rugosity is influenced by environmental and ecological factors, as well as geomorphic setting [90]. Local conditions, particularly wave energy, can shape reef structure, with more wave-exposed sites typically exhibiting lower structural complexity than protected reefs [91]. Wave energy affects reef structure by influencing coral community composition and CaCO_3_ framework production [51,92], as well as forming geomorphic features such as spur-and-groove systems [93]. This pattern was evident across our sites. In particular, the relatively sheltered Kāneʻohe Bay site was more rugose (1.28 ± 0.07) than higher energy sites such as Mākua (1.13 ± 0.03), Mokulēʻia (1.07 ± 0.03), and ʻEwa (1.13 ± 0.04) (Table 1). However, wave-exposed reefs can still maintain high coral cover and carbonate production despite flatter structural profiles when dominated by corals adapted to high-energy environments. For example, Kewalo had low rugosity (1.12 ± 0.02) similar to other wave-exposed sites, but supported relatively higher coral cover and gross carbonate production (Table 1). In comparison, Reef Runway, while also exposed to wave energy from the south, was the most rugose (1.43 ± 0.05) with high coral cover and CaCO_3_ production, further highlighting potential dissociation between rugosity and coral cover, and, consequently, between rugosity and gross carbonate production.

Underlying geomorphology also affects structural complexity. Reef habitats with high-relief substrates (e.g., spur and groove, rock and boulder) can yield rugosity values disproportionately higher than the CaCO_3_ framework formed by calcifiers, as seen when comparing Kāneʻohe Bay and Barbers Point. Rugosity at Barbers Point (1.34 ± 0.02) and Kāneʻohe Bay (1.28 ± 0.07) were not significantly different, yet Kāneʻohe Bay had > 45% higher hard coral cover and produced significantly higher CaCO_3_ per year (7.10 ± 0.81 kg m^-2^ yr^-1^) than Barbers Point (1.49 ± 0.32 kg m^-2^ yr^-1^). Benthic community composition may also contribute to an inconsistent relationship between rugosity and gross carbonate production. Generally, highly complex coral morphologies add significantly more three-dimensional structure and grow at faster rates (e.g., branching *Acropora = 8.64 ± 2.11 cm yr^-1^ linear extension) than less complex morphologies (e.g., encrusting Montipora* = 1.53 ± 0.36 cm yr^-1^ linear extension) [4,94]. However, this relationship is partly decoupled by the high cover of massive *Porites* across Oʻahu, which contributes substantially to colony-level calcification and site-level carbonate production, yet adds comparatively less three-dimensional complexity than branching or foliose morphologies. These findings indicate that rugosity alone may not reliably predict gross carbonate production, emphasizing the importance of considering geomorphic, environmental, and ecological controls on reef structural complexity.

### Bioerosion and relationships with structural complexity

Urchin erosion was the dominant contributor to site-level carbonate bioerosion at all sites except Mākua and Mokulēʻia. Despite this, neither urchin density or mean test size, when analyzed independently or in combination, predicted total bioerosion. This may reflect both limited statistical power (n = 7 sites) and contrasting size-density structures among sites that obscure simple linear relationships. Urchin bioerosion rates scale exponentially with test size and vary among species [95], meaning large individuals disproportionately drive total bioerosion rather than density alone. Site-level comparisons further reveal this decoupling of size and density (S4 Fig). For example, urchin density at Barbers Point (1.63 ± 0.07 ind. m^-2^) was approximately 3.4 times higher than at Reef Runway (0.37 ± 0.04 ind. m^-2^), yet mean total urchin bioerosion at Reef Runway (1.05 ± 0.52 kg CaCO_3_ m^-2^ yr^-1^) was nearly 50% higher than Barbers Point (0.6 ± 0.52 kg CaCO_3_ m^-2^ yr^-1^). This likely reflects differences in species composition and test-size structure among sites, with the presence of large-bodied *Echinothrix* spp*.* at Reef Runway (mean test size = 60.51 ± 4.15 mm), compared to smaller *Echinometra* spp., the dominant urchin surveyed at Barbers Point (mean test size = 32.25 ± 1.15 mm). Similarly, ‘Ewa had the largest urchins but only four individuals (mean test size = 80.97 ± 7.07 mm), resulting in relatively low total urchin bioerosion. In contrast, Kāneʻohe Bay exhibited relatively higher urchin bioerosion rates driven by higher density (1.27 ± 0.10 ind. m^-2^) of smaller urchins (mean test size = 32.71 ± 1.50 mm). Together, these patterns suggest that high densities of small urchins can compensate for reduced individual erosion rates, whereas low densities of large individuals may not generate equivalent site-level carbonate erosion.

A similar pattern was observed for parrotfishes. Abundance alone did not predict total carbonate bioerosion; instead, parrotfish biomass—which incorporates both size and abundance—was positively associated with total carbonate bioerosion, supporting it as a more ecologically meaningful predictor than abundance. Bioerosion rates of individual parrotfish increase with size, among other inherent characteristics (species, life-phase) [57,96,97]. As a result, reefs with lower abundances of parrotfish could still experience disproportionately higher rates of carbonate bioerosion if the parrotfish individuals surveyed were relatively large (i.e., higher biomass). For example, parrotfish abundance in Kāneʻohe Bay was ~ 4 times greater than at Reef Runway, yet parrotfish biomass at Reef Runway was ~ 1.6 times higher, resulting in higher estimated parrotfish bioerosion rates at Reef Runway (0.17 ± 0.06 kg CaCO_3_ m^-2^ yr^-1^) than in Kāneʻohe Bay (0.10 ± 0.04 kg CaCO_3_ m^-2^ yr^-1^). Overall, these findings suggest that bioerosion rates may be more strongly influenced by the presence of large, fast-eroding urchin and parrotfish individuals than by overall abundance alone.

Total carbonate bioerosion increased with structural complexity, although rugosity and bioerosion were not fully independent in this study because bioerosion calculations incorporated transect-level rugosity. This aligns with previous studies showing that more structurally complex reefs provide greater carbonate surface area, increasing habitat and food availability for bioeroders [3,98–104]. Interestingly, we did not observe a significant relationship between rugosity and bioeroder density or abundance, likely due to low sample sizes and overall low parrotfish presence. However, parrotfish biomass and erosion were positively correlated with rugosity, in line with previous studies [4,105,106], suggesting that reef complexity may be more strongly associated with bioeroder size structure than abundance or density alone. Similarly, urchin erosion was positively correlated with rugosity, and although urchin biomass was not directly quantified, larger mean test sizes were observed at more rugose sites. For example, Reef Runway, the most rugose site, had relatively moderate urchin density but supported urchins nearly twice the mean test size observed at Barbers Point and Kāneʻohe Bay (Table 1 and S4 Fig). These patterns highlight the disproportionate contribution of larger-bodied bioeroders to carbonate erosion and help explain the positive relationship between structural complexity and bioerosion, as bioerosion rate calculations are strongly influenced by bioeroder biomass. Many highly rugose sites with elevated urchin and parrotfish erosion were either naturally protected (Kāneʻohe Bay), or buffered by geomorphology and limited human access (e.g., Reef Runway and Barbers Point), conditions known to facilitate structural complexity and persistence of larger, more abundant bioeroders [3,99,104,107,108]. In contrast, endolithic (micro- and macroboring) bioerosion exhibited a generally negative relationship with hard coral cover across sites. Higher live coral cover reduces the availability of exposed carbonate substrate used to estimate endolithic bioerosion, constraining endolithic erosion rates. For example, Reef Runway had high rugosity and coral cover but relatively low endolithic bioerosion, consistent with substrate limitation.

Like carbonate production, limitation in location- and species-specific bioerosion data constrain the geographic and taxonomic resolution of our site-level bioerosion estimates. In general, the published urchin erosion rates used to calculate total urchin bioerosion [20] are relatively sparse (27 reported rates), and are largely derived from studies concentrated in the Eastern Pacific (8 studies) and Indian Ocean (7 studies) regions, with few conducted within the Western and Central Pacific Islands region (4 studies). Parrotfish bioerosion metrics are similarly limited in the Pacific, despite recent synthesis of allometric foraging equations [57]. Additionally, macrobioerosion and microbioerosion rates are typically calculated using Indo-Pacific average rates [20] and percent cover of non-coral erodible substrate. Endolithic erosion can occur beneath live coral tissue [109,110], but is not explicitly accounted for in these calculations. Incorporating endolithic erosion within living carbonate framework and expanding bioerosion measurements across taxa in the Pacific Islands region would further refine total carbonate bioerosion assessments.

### Application of carbonate budgets data and future directions

Climate change, ocean acidification, and other environmental stressors threaten to reduce percent cover, diversity, growth rates, and structural complexity of coral reef calcifiers and accelerate rates of carbonate erosion over the next several decades [36,111–113]. Resulting changes in the abundance and activity of habitat-altering taxa can, in turn, drive significant changes in reef carbonate budgets. On Oʻahu, we found massive *Porites,* encrusting *Montipora*, and branching *Pocillopora* to be significant contributors to coral cover, carbonate production, and reef structural complexity. These genera have already been impacted by recent thermal stress events, with branching *Pocillopora* observed to be most vulnerable to bleaching of all Hawaiian coral genera [75]. Further declines in the cover of these corals, coupled with decreasing calcification rates predicted under ocean acidification [114], could decrease the gross carbonate production and structural complexity of Oʻahu reefs. Such changes may also shift coral community composition towards more opportunistic, non-reef building taxa. Additionally, loss of structurally complex habitat could lead to changes in bioeroder populations, further influencing reef carbonate budgets. For reefs already at the threshold for maintaining net positive carbonate production (e.g., ʻEwa, Mākua, Barbers Point), ecological changes such as these have the potential to shift a reef into a state of net erosion if declines in gross carbonate production rates are greater than the reduction in bioerosion rates. Strategic coral restoration efforts at these sites may serve as an important tool for maintaining positive carbonate production and preventing future net erosional trajectories. With rapidly changing climate conditions and reef ecological states, the ability to understand how reef communities may be changing and subsequently affecting net carbonate budgets over time is essential.

## Supporting information

S1 Fig Comparisons of average coral linear growth rates (cm yr^-1^ ± CI) derived from studies throughout the entire Indo-Pacific region (*ReefBudget*) versus the Pacific Islands region. Genus-morphology groups are represented as genus code-morphology using the following abbreviations: Genus codes: FUSP = Fungia, LEPT = Leptastrea, LESP = Leptoseris, MOSP = Montipora, PAVS = Pavona, POCS = Pocillopora, POSP = Porites; Morphology: FR = free-living, EM = encrusting, FO = foliose, BR = branching, MD = mounding (massive), ML = mounding-lobate. Confidence intervals (CI) are zero, when only 1 study was used as representative of that coral taxa growth rate within the region defined.
(TIF)

S2 Fig Total contributions of dominant Oʻahu coral taxa (morphology-genus), summed across sites, to A) total gross carbonate production ± standard error (SE), and B) total percent coral cover ± SE. (TIF)

S3 Fig Contributions of dominant Oʻahu coral taxa (morphology-genus) to A) site-level gross carbonate production ± standard error (SE), and B) site-level percent coral cover ± SE. Within bar colors represent contributions from each coral morphology-genus group. “Other” = branching *Porites*, encrusting *Pavona*, massive *Pavona*, encrusting *Leptastrea*, other encrusting corals, and free-living corals.
(TIF)

S4 Fig Scatter plot showing site-level mean urchin test size (mm ± SE) and urchin density (individuals m^-2^ ± SE) for the seven Oʻahu survey sites. (TIF)

S1 Table *ReefBudget* and NCRMP benthic survey taxonomic codes. (DOCX)
