# Supplementary material for: A Pacific carbonate budgets approach reveals overall net positive production on O‘ahu, Hawai‘i
Source: PLoS One. 2026 Aug 12;21(8):e0354186. doi: 10.1371/journal.pone.0354186 (PMC13465867; doi:10.1371/journal.pone.0354186)
Supplement: Supplementary file 1 [file pone.0354186.s005.docx]

| **Coral taxa** | **Morphology** | **ReefBudget (genus + morphology)** | **NCRMP (species or genus + morphology)** |
| --- | --- | --- | --- |
| *Acropora sp.* | table | ACRT | ACSP -TB |
| *Acropora cytherea* | table |  | ACYT |
| *Acropora paniculata* | table |  | APAN |
| *Acropora sp.* | branching | ACRB | ACSP-BR |
| *Acropora gemmifera* | branching |  | AGEM |
| *Acropora humilus* | branching |  | AHUM |
| *Coscinaraea sp.* | encrusting-mounding | COSE | COSP -EM |
| *Cycloseris sp* | free-living | CYC | CYSP -FL |
| *Cyphastrea sp.* | encrusting-mounding | CYPE | CYPS -EM |
| *Diaseris sp.* | free-living | DIA | DIAS-FL |
| *Fungia sp.* | free-living | FUN | FUSP-FL |
| *Gardineroseris sp.* | mounding | GARM | GASP-MD |
| *Gardineroseris planulata* | mounding |  | GPLA |
| *Leptastrea sp.* | encrusting-mounding | LEPE | LEPT-EM |
| *Leptastrea bewickensis* | encrusting-mounding |  | LBEW |
| *Leptastrea purpurea* | encrusting-mounding |  | LPUR |
| *Leptoseris sp.* | foliose | LESF | LESP-FO |
| *Montipora sp.* | branching | MOPB | MOSP-BR |
| *Montipora capitata* | branching |  | MCAP |
| *Montipora incrassata* | branching |  | MINC |
| *Montipora sp.* | encrusting-mounding | MOPE | MOSP-EM |
| *Montipora capitata* | encrusting-mounding |  | MCAP |
| *Montipora dilatata* | encrusting-mounding |  | MDIL |
| *Montipora flabelatta* | encrusting-mounding |  | MFLA |
| *Montipora patula* | encrusting-mounding |  | MPAT |
| *Montipora turgescens* | encrusting-mounding |  | MTUR |
| *Montipora sp.* | foliose | MOPF | MOSP-FO |
| *Pavona sp.* | encrusting-mounding | PAVE | PAVS-EM |
| *Pavona chiriquiensis* | encrusting-mounding |  | PCHI |
| *Pavona maldivensis* | encrusting-mounding |  | PMAL |
| *Pavona varians* | encrusting-mounding |  | PVAR |
| *Pavona sp.* | mounding | PAVM | PAVS-MD |
| *Pavona duerdeni* | mounding-lobate |  | PDUE |
| *Pocillopora sp.* | branching | POCB | POCS-BR |
| *Pocillopora damicornis* | branching |  | PDAM |
| *Pocillopora grandis* | branching |  | PGRA |
| *Pocillopora ligulata* | branching |  | PLIG |
| *Pocillopora meandrina* | branching |  | PMEA |
| *Porites sp.* | branching | PORB | POSP-BR |
| *Porites compressa* | branching |  | PCOM |
| *Porites sp.* | mounding | PORM | POSP-MD |
| *Porites lobata* | mounding |  | PLOB |
| *Porites lutea* | mounding |  | PLUT |
| *Porites sp.* | encrusting-mounding | PORE | POSP |
| *Porites bernardi* | encrusting-mounding |  | PBER |
| *Porites lichen* | encrusting-mounding |  | PLIC |
| *Porites sp.* | laminar-columnar | PORC | POSP-LC |
| *Porites monticulosa* | laminar-columnar |  | PMON |
| *Porites rus* | laminar-columnar |  | PRUS |
| *Psammocora sp.* | encrusting-mounding | PSAE | PSSP-EM |
| *Psammocora nierstraszi* | encrusting-mounding |  | PNIE |
| *Psammocora profundacella* | encrusting-mounding |  | PPRO |
| *Psammocora stellata* | encrusting-mounding |  | PSTE |
